# Regulation of dopamine-dependent transcription and cocaine action by *Gadd45b*

**DOI:** 10.1038/s41386-020-00828-z

**Published:** 2020-09-14

**Authors:** Morgan E. Zipperly, Faraz A. Sultan, Guan-En Graham, Andrew C. Brane, Natalie A. Simpkins, Nancy V. N. Carullo, Lara Ianov, Jeremy J. Day

**Affiliations:** 1 Department of Neurobiology, University of Alabama at Birmingham, Birmingham, AL 35294 USA; 2 Civitan International Research Center, University of Alabama at Birmingham, Birmingham, AL 35294 USA

**Keywords:** Addiction, Motivation

## Abstract

Exposure to drugs of abuse produces robust transcriptional and epigenetic reorganization within brain reward circuits that outlives the direct effects of the drug and may contribute to addiction. DNA methylation is a covalent epigenetic modification that is altered following stimulant exposure and is critical for behavioral and physiological adaptations to drugs of abuse. Although activity-related loss of DNA methylation requires the *Gadd45* (Growth arrest and DNA-damage-inducible) gene family, very little is known about how this family regulates activity within the nucleus accumbens or behavioral responses to drugs of abuse. Here, we combined genome-wide transcriptional profiling, pharmacological manipulations, electrophysiological measurements, and CRISPR tools with traditional knockout and behavioral approaches in rodent model systems to dissect the role of *Gadd45b* in dopamine-dependent epigenetic regulation and cocaine reward. We show that acute cocaine administration induces rapid upregulation of *Gadd45b* mRNA in the rat nucleus accumbens, and that knockout or site-specific CRISPR/Cas9 gene knockdown of *Gadd45b* blocks cocaine conditioned place preference. In vitro, dopamine treatment in primary striatal neurons increases *Gadd45b* mRNA expression through a dopamine receptor type 1 (DRD1)-dependent mechanism. Moreover, shRNA-induced *Gadd45b* knockdown decreases expression of genes involved in psychostimulant addiction, blocks induction of immediate early genes by DRD1 stimulation, and prevents DRD1-mediated changes in DNA methylation. Finally, we demonstrate that *Gadd45b* knockdown decreases striatal neuron action potential burst duration in vitro, without altering other electrophysiological characteristics. These results suggest that striatal *Gadd45b* functions as a dopamine-induced gene that is necessary for cocaine reward memory and DRD1-mediated transcriptional activity.

## Introduction

Addiction is an increasingly prevalent problem in the United States, associated with progressively higher rates of morbidity and mortality. Experience with drugs of abuse results in significant transcriptional and epigenetic alterations in the nucleus accumbens (NAc) that support both synaptic and behavioral plasticity, outlasting the direct effects of the drug and contributing to the development of addiction [1–4]. Despite their various mechanisms of action, one common feature of many drugs of abuse is that they act upon the mesocorticolimbic dopamine (DA) system, which includes the ventral tegmental area (VTA), the NAc, and the prefrontal cortex [2, 5]. Addictive substances exert their rewarding effects in part by elevating DA concentrations in the NAc, a brain region integral to reward learning, as well as the development and maintenance of addiction [6–8]. This increase in DA modifies neuronal function by usurping mechanisms underlying adaptive forms of learning, activating signaling cascades and transcriptional programs that contribute to the reinforcing properties of drugs of abuse and leading to drug-evoked synaptic plasticity [2, 6, 8–12].

Epigenetic mechanisms include post-translational modification of histone proteins and methylation of cytosine-phospho-guanine (CpG) dinucleotides within DNA, which alter chromatin structure and transcription factor activity to modulate gene expression [1, 4, 13]. DNA methylation is catalyzed by DNA methyltransferases (DNMTs), and promoter hypermethylation is traditionally associated with transcriptional silencing [4]. Previous work has demonstrated upregulation of DNMT3A and DNMT3B (proteins which are required for de novo methylation) in the NAc 4 hr following acute cocaine treatment; by 24 hr, these levels decreased below baseline [4, 14, 15]. This biphasic regulation of DNMT3A at 4 hr and 24 hr was also observed after 7 days of repeated cocaine administration [15]. However, a more recent study examining methylation machinery in the NAc found decreased DNMT3A 30 minutes following acute cocaine treatment and increased levels of the protein at 24 hr [16]. Yet another study demonstrated upregulation of *Dnmt3a2*, but not *Dnmt3a1*, in the NAc shell following acute cocaine experience [17]. These data suggest complex temporal dynamics in the regulation of DNA methylation machinery. In rats that chronically self-administered cocaine, DNMT3A expression in the NAc was downregulated 24 hr after the last cocaine infusion [15]. Intriguingly, both DNMT inhibition and chronic methyl supplementation blocked the expression of cocaine-induced locomotor sensitization, despite their opposing effects on DNA methylation [4, 14]. Furthermore, intra-cranial delivery of the DNA methylation inhibitor RG108 resulted in enhanced cocaine conditioned place preference (CPP) when targeted to the NAc [15], but impaired associative reward learning when delivered to the VTA [18]. Conversely, DNMT3A viral overexpression in the NAc attenuated the expression of cocaine-paired place preference, whereas NAc DNMT3A knockdown increased cocaine CPP [15]. Following cocaine reward, differential hypo- and hypermethylation events have been observed at specific gene loci at different timepoints [4, 14, 18, 19]. In a rat model of incubation of cocaine craving, NAc-specific DNMT inhibition blocked reinstatement of cue-induced cocaine-seeking behavior after 30 days withdrawal, and this effect was associated with long-lasting changes in DNA methylation at gene promoters [19]. Together, these findings suggest that DNA methylation dynamics are critically important for drug- and reward-related behaviors (see [20] for a comprehensive review) and highlight the importance of identifying the underlying molecular mechanisms that contribute to these changes.

Although numerous studies have demonstrated that DNA methylation marks are relatively stable and mediate long-term transcriptional regulation, the specific mechanisms that regulate CpG demethylation remain unclear. Previous studies have characterized the role of ten-eleven translocation (TET) enzymes in the conversion of 5-methylcytosine (5mC) to 5-hydroxymethylcytosine (5hmC) and additional oxidation that results in base excision and replacement [21–23]. In the brain, *Gadd45b*, a member of the Growth arrest and DNA-damage-inducible gene family, is associated with decreased CpG methylation in gene regulatory regions and is critical for activity-dependent DNA demethylation, potentially working in concert with TET and thymine-dependent glycosylase proteins [21, 24–27]. Moreover, *Gadd45b* is upregulated in response to neuronal activity in multiple brain regions and plays a key role in experience-dependent learning [24, 25, 28, 29]. These results demonstrate that *Gadd45b* may link experience-dependent neuronal activity and downstream regulation of DNA methylation states.

However, despite enrichment of *Gadd45b* in the striatum [28, 30, 31] and clear links between drug-related transcriptional changes and DNA methylation, the role of *Gadd45b* in this process has not been examined. Here, we report that *Gadd45b* functions as an immediate early gene (IEG) in the rat NAc following acute cocaine reward or exposure to cocaine-paired contexts in vivo. Furthermore, *Gadd45b* disruption attenuates cocaine-paired place preference, suggesting that *Gadd45b* action is required for cocaine reward memory. Using a primary rat striatal neuron culture system, we demonstrate that DA-induced increases in *Gadd45b* require DRD1 activation, mitogen-activated protein kinase (MEK) signaling, and cAMP response element binding protein (CREB). Furthermore, knockdown of *Gadd45b* in vitro results in the downregulation of IEGs and of genes implicated in dopaminergic synapse function and drug addiction and also abolishes changes in DNA methylation status following DRD1 activation. Intriguingly, although we observe significant transcriptional and behavioral alterations following *Gadd45b* manipulation, electrophysiological responses to DRD1 stimulation remain unchanged. Together, these results characterize *Gadd45b* as a DA-induced IEG in the striatum that is critical for transcriptional and behavioral effects of cocaine.

## Materials and methods

Complete details for all “Materials and methods” is provided in Supplemental Materials.

### Animals

All experiments were performed in accordance with the University of Alabama at Birmingham Institutional Animal Care and Use Committee. Sprague–Dawley timed pregnant dams and adult male rats (90–120 days old) were purchased from Charles River Laboratories (Wilmington, MA, USA). *Gadd45b* knockout mice were bred at the University of Alabama at Birmingham on a B6:129VJ background, as described previously [28, 32].

### Neuronal cell cultures

Primary rat striatal cell cultures were generated from E18 striatal tissue as described previously [33–35].

### RNA extraction and RT-qPCR

Total RNA was extracted (RNAeasy kit, Qiagen, Hilden, Germany) and reverse-transcribed (iScript cDNA Synthesis Kit, Bio-Rad, Hercules, CA, USA). cDNA was subject to RT-qPCR for genes of interest, as described previously [34, 35]. A list of PCR primer sequences is provided in Supplemental Table S1.

### CRISPR/Cas9 and RNAi construct design

CRISPR and shRNA constructs for editing or knockdown of *Gadd45b* were delivered using second-generation lentiviral expression vectors. Cas9 and CRISPR single guide RNAs (sgRNAs) were expressed using a modified version of the lentivirus-compatible expression vector lentiCRISPR v2 [36], which was a gift from Feng Zhang (Addgene plasmid #52961). *Gadd45b*-specific sgRNA targets were designed using online tools provided by the German Cancer Research Center (http://www.e-crisp.org/E-CRISP/). To ensure specificity, CRISPR RNA (crRNA) sequences were analyzed with Cas-OFFinder [37]. Critically, the *Gadd45b* crRNA sequence did not have any identical matches elsewhere in the rat genome, had no single base mismatches, and had only one two-nucleotide mismatch sequence, which was not located in a gene (Table S1).

ShRNAs were designed using the Broad Institute Genetic Perturbation Platform web portal. A pLKO.1-TRC vector (a gift from David Root; Addgene plasmid #10879) [38] was cloned into an expression vector with an mCherry reporter (Addgene plasmid #114199) [33] to generate distinct U6-shRNA and EF1α-mCherry expression cassettes. A list of the sgRNA and shRNA target sequences is provided in Table S1.

### Lentivirus production

Viruses were generated as described previously [33, 35].

### Multielectrode array recordings

Single-unit electrophysiological activity was recorded using an Axion Maestro Pro recording system (Axion Biosystems). E18 rat primary striatal neurons were seeded in 48-well MEAs at 30,000 cells/well. Each MEA well within the 48-well plate contained 16 extracellular recording electrodes and a ground electrode. Further details provided in Supplemental Materials.

### RNA sequencing

RNA was extracted, purified (RNeasy, Qiagen), and DNase-treated for three biological replicates per experimental condition. RNA quality was determined on the BioAnalyzer 2100 (Agilent Technologies, Wilmington, DE, USA). RNA sequencing libraries were created using the NEBNext Ultra II Directional RNA-Seq library kit (NEB, Ipswich, MA, USA) according to manufacturer’s recommendations. The resulting libraries underwent sequencing (75 bp paired-end directional reads; 28.9–48.7 million reads/sample) on an Illumina NextSeq 500 sequencing platform using standard techniques. Details on RNA-seq data analysis are provided in Supplemental Materials.

### Reduced representation bisulfite sequencing (RRBS)

Genomic DNA from ~250,000 neurons per sample (*n* = 3 samples per group) was extracted and purified (DNeasy Blood and Tissue DNA extraction kit, Qiagen) prior to RRBS (Ovation RRBS Methyl-Seq System, NuGen, #0353), used according to manufacturer’s instructions. Bisulfite-converted DNA libraries underwent sequencing (75 bp single-end reads; ∼28.7–35.4 million reads/sample) on an Illumina sequencing platform (NextSeq 500). See Supplemental Materials for details on RRBS data analysis.

### Acute cocaine locomotor testing and cocaine sensitization

For open-field locomotor testing, naive male rats (*n* = 12 per group) were given i.p. injections of saline on days 1 and 2 immediately before 30 min of locomotor testing. On days 3 and 10, half of the rats were given an i.p. injection of 10 mg/kg cocaine, and the other half received i.p. injections of saline. Locomotor activity was monitored in a 43 cm × 43 cm plexiglass locomotor activity chamber (Med Associates, Inc., St. Albans, VT, USA) with opaque white wall covering and an open top.

### CPP testing

CPP testing was completed in a three-chamber apparatus with guillotine doors (Med Associates, Inc.). For CPP testing in transgenic *Gadd45b* knockout mice, naive male mice were placed in the central compartment on the first day of testing (i.e., pre-test) and were permitted to explore all three chambers of the CPP apparatus during a 20 min session. On days 2 and 4, mice were given an i.p. injection of saline immediately prior to being placed in the initially preferred chamber for the 20 min conditioning session. On days 3 and 5, mice were given an i.p. injection of 10 mg/kg cocaine before being placed in the initially non-preferred chamber for the 20 min conditioning session. On day 6 (post test), mice were again placed in the central compartment and allowed to freely move between all three chambers.

CPP testing in adult male rats began 2 weeks following viral infusion surgeries. Days 1–6 of testing were identical to CPP testing in transgenic mice. However, this schedule was repeated in rats on days 7–10, increasing the cocaine dose to 20 mg/kg for cocaine conditioning on days 8 and 10. On day 11, rats underwent a second post test to measure cocaine place preference. Testing and conditioning sessions lasted 30 min.

## Results

### *Gadd45b* is induced by acute cocaine and cocaine-paired contexts

**Fig. 1:**
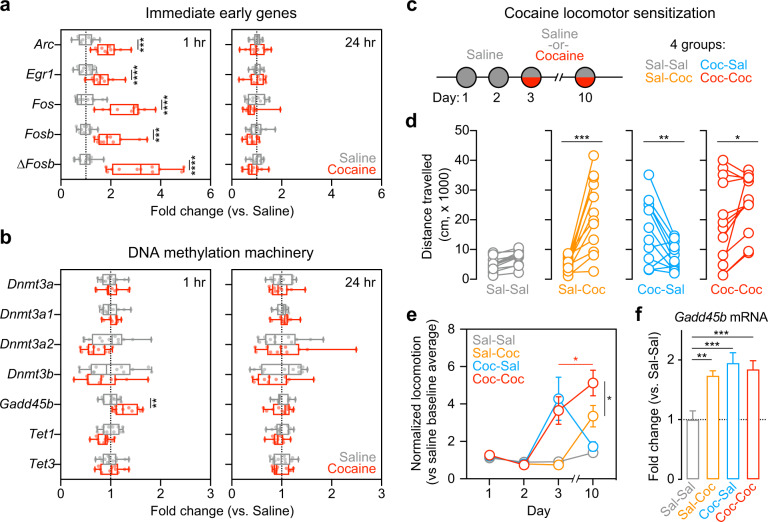
Exposure to acute cocaine increases expression of immediate early genes and *Gadd45b* in vivo. **a** Adult male rats were administered a single i.p. injection of saline or 10 mg/kg cocaine and returned to their home cage. NAc tissue was collected bilaterally at either 1 hr or 24 hr post injection. RT-qPCR reveals significant increases in immediate early gene (IEG) expression at 1 hr in cocaine-treated rats compared with saline controls. No changes were observed at 24 hr. **b** Of genes involved in activity-dependent DNA methylation and demethylation processes, only *Gadd45b* mRNA was significantly increased 1 hr following acute exposure to cocaine. There were no significant changes observed at 24 hr. **c** To examine cocaine locomotor sensitization, all rats were administered saline on days 1 and 2. On days 3 and 10, half received a 10 mg/kg cocaine injection. This yielded four groups. **d** Distance traveled was significantly increased in animals that received cocaine either on day 3 or on day 10. Animals who received two consecutive cocaine injections on day 3 and day 10 exhibited further increases in locomotor activity. **e** Locomotor sensitization was only observed in animals that received cocaine on both day 3 and day 10. Distance traveled on days 3 and 10 was normalized within group to mean locomotion of baseline days 1 and 2. **f** NAc tissue was collected bilaterally 1 hr post injection on day 10 for RT-qPCR analysis. *Gadd45b* mRNA was significantly increased in all groups with cocaine experience. All data are expressed as mean ± s.e.m. *p < 0.05, **p < 0.01, ***p < 0.001, and ****p < 0.0001 for indicated comparisons.

To examine the effects of cocaine on *Gadd45b* expression in vivo, we collected NAc tissue of adult naive male rats at 1 hr and 24 hr following treatment with either cocaine (10 mg/kg, i.p.; *n* = 11) or saline (*n* = 12). As expected, mRNA for several classic IEGs (*Arc*, *Egr1*, *Fos*, *Fosb*, and *ΔFosb*) was transiently increased 1 hr after cocaine injection (multiple *t* tests, *Arc t*_(21)_ = 5.188, adjusted *p* = 0.000116; *Egr1 t*_(21)_ = 4.078, adjusted *p* = 0.000539; *Fos t*_(21)_ = 6.552, adjusted *p* = 0.000008; *Fosb t*_(21)_ = 4.919, adjusted *p* = 0.000145; *ΔFosb t*_(21)_ = 6.581, adjusted *p* = 0.000008), with a return to baseline at 24 hr (Fig. 1a). Similarly, RT-qPCR for *Gadd45b* revealed significant upregulation at 1 hr following cocaine treatment (multiple *t* tests, *Gadd45b t*_(21)_ = 3.651, adjusted *p* = 0.010389), but not at 24 hr (Fig. 1b). Given that GADD45 proteins have been implicated in active demethylation [26, 27], we also measured expression of other genes involved in DNA methylation, such as *Dnmt3a*, *Dnmt3a1*, *Dnmt3a2*, *Dnmt3b*, *Tet1*, and *Tet3* (Fig. 1b). However, expression of these genes did not differ between cocaine- and saline-treated rats at either timepoint.

In order to better characterize how cocaine experience affects *Gadd45b* expression, we used a locomotor sensitization paradigm [39] in which rats received an injection of saline on days 1–2 and an injection of either saline or cocaine (10 mg/kg) on days 3 and/or 10 (Fig. 1c, *n* = 12/group). Acutely, rats that received cocaine displayed heightened locomotor activity compared with saline controls (Fig. 1d; paired *t* tests, Sal-Coc *t*_(11)_ = 5.094, *p* = 0.0003; Coc-Sal *t*_(11)_ = 3.219, *p* = 0.0082; Coc-Coc *t*_(11)_ = 2.268, *p* = 0.0445), and locomotor sensitization was only observed in rats that received cocaine on both day 3 and day 10 (Fig. 1e, two-way analysis of variance (ANOVA, *F*_(9,132)_ = 9.714, *p* < 0.0001 for day × group interaction; Tukey’s post hoc tests, *p* < 0.05 for indicated comparisons). NAc tissue was collected 1 hr following treatment on day 10, and RT-qPCR revealed that *Gadd45b* mRNA was upregulated in all groups with cocaine experience (Fig. 1f, one-way ANOVA, *F*_(3,44)_ = 8.9925, *R*^*2*^ = 0.38, *p* < 0.0001; Tukey’s post hoc tests, Sal-Coc *p* < 0.01; Coc-Sal *p* < 0.001, Coc-Coc *p* < 0.001), suggesting that *Gadd45b* is induced by cocaine and cocaine-paired environments, independent of the acute psychostimulant action of the drug. These findings are in line with literature demonstrating that drug-associated cues promote DA release [8, 40, 41], as well as previous evidence that Drd1-medium spiny neurons (MSNs) are specifically activated by re-entry to a cocaine-paired context [42].

### Striatal *Gadd45b* is necessary for cocaine reward memory

**Fig. 2:**
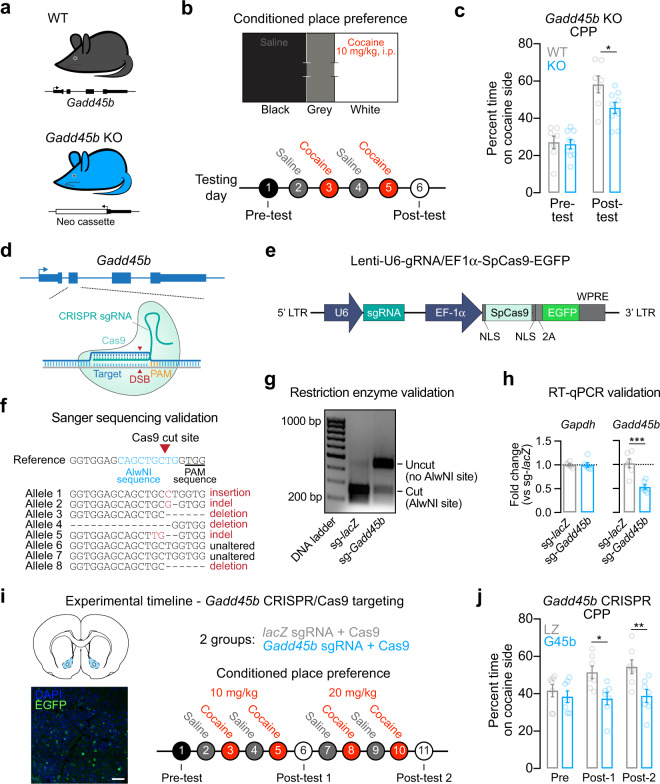
Efficient gene knockdown of *Gadd45b* attenuates cocaine reward memory. **a** Male wild-type and mutant *Gadd45b* knockout mice between 2 and 6 months of age were used for behavioral testing. **b** Conditioned place preference (CPP) apparatus and experimental timeline for CPP testing. **c** Cocaine-paired place preference was significantly attenuated in *Gadd45b* mutant mice. **d** Targeting strategy to induce insertion/deletion mutations in *Gadd45b*. CRISPR guide RNAs (gRNAs) were engineered to target exon 2 of *Gadd45b*. **e** Illustration of lentivirus construct designed to express gRNA (driven by the U6 promoter) and the Cas9 nuclease (driven by the Ef1α promoter), with bicistronic expression of EGFP. **f** Sanger sequencing of individual genomic DNA clones reveals CRISPR-mediated mutation in six of eight alleles (75% efficiency) following lentiviral transduction. Primary striatal neuron cultures were transduced with lentivirus at DIV7 and genomic DNA was extracted at DIV11. **g** Genomic DNA restriction digest at AlwNI site (which is mutated by Cas9 editing) reveals almost complete digestion in *lacZ* gRNA control, but loss of digestion when AlwNI site has been mutated in *Gadd45b* gRNA group. Data from primary striatal neuron cultures. **h** RT-qPCR validation demonstrates robust knockdown of *Gadd45b* mRNA following Cas9 targeting in striatal neuron cultures. Remaining mRNA contains insertions/deletions resulting in premature translation termination or dysfunctional protein. **i** Experimental timeline for CRISPR/Cas9 viral transduction and cocaine CPP testing. Lentiviral constructs targeting *lacZ* or *Gadd45b* were bilaterally injected into the NAc core 2 weeks prior to behavioral testing. Inset image shows EGFP expressed from SpCas9-2A-EGFP cassette in a representative NAc section. Scale bar = 50 µm. **j** Rats that received the *Gadd45b*-targeted CRISPR/Cas9 construct failed to form a place preference to cocaine at both 10 mg/kg and 20 mg/kg. All data are expressed as mean ± s.e.m. *p < 0.05, **p < 0.01, and ***p < 0.001 for indicated comparisons.

Given that *Gadd45b* is important for learning and memory [28, 29] and is upregulated following acute cocaine experience, we next aimed to determine whether *Gadd45b* expression is necessary for cocaine reward memory. To do this, we employed a CPP paradigm, which is commonly used to examine drug-associated memories and the rewarding properties of drugs of abuse. In this assay, drug-naive animals freely explore a three-chambered apparatus on day 1 (Fig. 2b) to assess initial preference for a particular context. Following four days of conditioning, animals are again allowed to freely explore the apparatus on day 7 in the absence of the drug to determine whether they exhibit a preference for the drug-paired context. Following conditioning, wild-type mice (WT, *n* = 7) formed a normal CPP response for the cocaine-paired chamber. However, this cocaine-paired place preference was attenuated in *Gadd45b* knockout mice (KO, *n* = 9; two-way ANOVA, *F*_(1,14)_ = 6.232, *p* = 0.0256 for group comparison; Bonferroni’s post hoc multiple comparisons, post test WT vs KO *p* < 0.05) despite similar time spent on the cocaine-paired side during the pre-test, suggesting a deficit in cocaine reward memory (Fig. 2a–c).

We next sought to characterize the specific role of *Gadd45b* in the NAc. We repeated this behavioral assay using *Gadd45b*-targeted CRISPR/Cas9 gene editing in the ventral striatum in rats in order to produce site-specific insertion/deletion events that render dysfunctional GADD45B protein. We designed lentiviral constructs expressing the Cas9 nuclease and CRISPR sgRNA targeted to exon 2 of *Gadd45b* (Fig. 2d, e) and validated successful gene editing in vitro using three complementary approaches. Sanger sequencing revealed *Gadd45b* locus mutations in six out of eight alleles following transduction with CRISPR constructs, indicating 75% editing efficiency (Fig. 2f). Furthermore, restriction enzyme digest of genomic DNA at an AlwNI site located within the sgRNA sequence (overlapping the Cas9 cut site) revealed near-complete digestion in the *lacZ* non-targeting sgRNA control, indicative of an intact AlwNI site. In contrast, the *Gadd45b* sgRNA group lacked AlwNI-mediated digestion, confirming Cas9-driven mutation at the AlwNI site (Fig. 2g). As a final validation measure, RT-qPCR for *Gadd45b* mRNA indicated significant reduction in *Gadd45b* sgRNA-targeted cells (as compared with the *lacZ* sgRNA control, *n* = 6/group), demonstrating that gene editing was sufficient to reduce total levels of *Gadd45b* expression (Fig. 2h, unpaired *t* test, *t*_(10)_ = 4.615, *R*^*2*^ = 0.68, *p* = 0.001). Adult male rats (*n* = 7–8 per group) underwent stereotaxic surgery to infuse the CRISPR/Cas9 + sgRNA lentivirus bilaterally targeting the NAc core. After allowing 2 weeks for viral expression, CPP testing began (Fig. 2i). Whereas both groups exhibited a similar preference prior to cocaine pairing, only the *lacZ*-targeted rats developed a place preference for cocaine at the two doses tested (Fig. 2j, two-way ANOVA, *F*_(1,12)_ = 8.053, *p* = 0.0150 for group comparison, Bonferroni’s post hoc multiple comparisons). Rats that received the *Gadd45b*-targeted CRISPR/Cas9 gene editing constructs failed to develop a cocaine-paired place preference at both 10 mg/kg (*p* < 0.05) and 20 mg/kg (*p* < 0.01), suggesting that *Gadd45b* in the NAc is necessary for cocaine reward memory.

### DA-induced upregulation of *Gadd45b* requires DRD1 signaling pathways

**Fig. 3:**
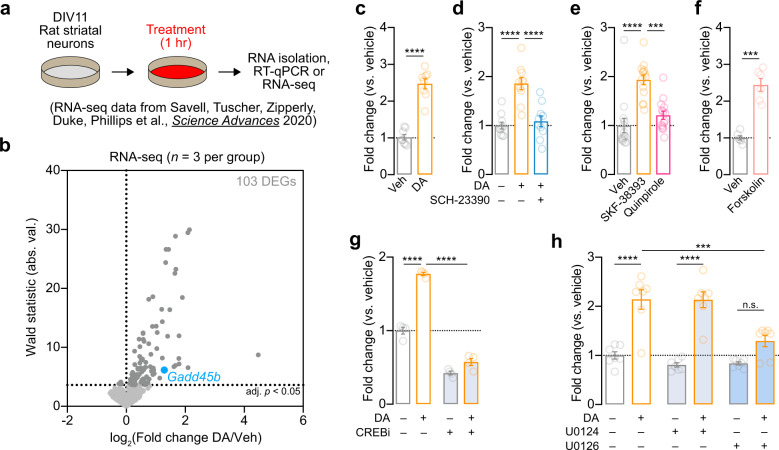
Induction of *Gadd45b* mRNA by dopamine requires DRD1 dopamine receptors and CREB activation in vitro. **a** Illustration of experimental design using primary rat striatal culture system. **b** Unbiased identification of DA-induced DEGs with RNA-seq reveals *Gadd45b* as one of 103 DA-induced IEGs. **c** Cells were treated with DA (1 µM) for 1 hr prior to RNA isolation and RNA-seq. **c** RT-qPCR validation of DA-induced increase in *Gadd45b* mRNA. **d** DA-induced increases in *Gadd45b* mRNA are blocked by co-treatment with the DRD1 receptor antagonist SCH-23390 (1 µM). **e** *Gadd45b* mRNA induction is mimicked by DRD1 receptor agonist SKF-38393 (1 µM), but not the D2/D3 receptor agonist Quinpirole (1 µM). **f**, *Gadd45b* mRNA is induced by the adenylyl cyclase activator Forskolin (20 µM). **g** CREB inhibition with 666-15 (1 µM) blocks baseline and DA-induced increases in *Gadd45b* mRNA. **h** MEK inhibition with U0126 (1 µM) prevents DA-induced increases in *Gadd45b* mRNA. U0124 (1 µM) is an inactive analog of U0126 and was included as a negative control. ***p < 0.001 and ****p < 0.0001 for indicated comparisons.

In order to further examine how DA regulates *Gadd45b* expression, we first used a well-established and highly controllable rat primary striatal neuron culture system [33–35] to examine DA-dependent *Gadd45b* induction (Fig. 3a). DIV11 striatal cultures were treated with 1 µM DA for 1 hr, a treatment which closely models both the increase in DA concentration and the temporal dynamics of DA changes in the striatum in vivo following acute cocaine exposure [6, 7, 43]. Using a recently published data set in which RNA-seq was performed on striatal neurons 1 hr after DA treatment [35], we found that *Gadd45b* was identified as one of only 100 mRNAs increased by DA (Fig. 3b). To validate this finding, as well as dissect the relevant DA receptor systems integral to *Gadd45b* action, we performed RT-qPCR on DA-treated striatal cultures (Fig. 3a). In agreement with RNA-seq results, we found that acute DA treatment significantly increased *Gadd45b* mRNA (Fig. 3c, *n* = 8/group; unpaired *t* test, *t*_(14)_ = 9.377, *R*^*2*^ = 0.86, *p* < 0.0001). DA-induced increases in *Gadd45b* mRNA were blocked in the presence of the DRD1 antagonist SCH-23390 (1 µM; Fig. 3d; *n* = 10/group; one-way ANOVA, *F*_(2,27)_ = 20.78, *R*^*2*^ = 0.61, *p* < 0.0001; Tukey’s post hoc test, *p* < 0.0001 for indicated comparisons). Similarly, treatment with DRD1 agonist SKF-38393 (1 µM), but not DRD2/DRD3 agonist quinpirole (1 µM), significantly increased *Gadd45b* expression compared with vehicle-treated controls (Fig. 3e; *n* = 14/group; one-way ANOVA, *F*_(2,39)_ = 18.73, *R*^*2*^ = 0.49, *p* < 0.0001; Tukey’s post hoc test *p* < 0.001 for indicated comparisons). *Gadd45b* mRNA was also elevated following stimulation with Forskolin (20 µM; *n* = 6/group), an activator of adenylyl cyclase that mimics DRD1-mediated G-protein coupled receptor signaling pathways (Fig. 3f, unpaired *t* test with Welch’s correction, *t*_(6, 198)_ = 7.781, *R*^*2*^ = 0.91, *p* = 0.0002). In order to better characterize the signaling cascades necessary for DA-induced increases in *Gadd45b*, we next co-treated striatal cultures with DA and either a CREB inhibitor (666-15, 1 µM) or a MEK inhibitor (U0126, 1 µM; *n* = 4/group). CREB inhibition decreased *Gadd45b* mRNA both at baseline and following DA treatment (Fig. 3g; two-way ANOVA, *F*_(1,12)_ = 560.3, *p* < 0.0001 for main effect of CREB inhibition; Sidak’s post hoc tests, *p* < 0.0001 for indicated comparisons). Similarly, MEK inhibition blocked baseline and DA-induced *Gadd45b* expression, compared with vehicle- or U0124-treated controls (Fig. 3h; *n* = 7/group; two-way ANOVA, *F*_(2,36)_ = 26.51, *p* = 0.0004 for main effect of MEK inhibition; Sidak’s post hoc tests, ****p < 0.0001 and ***p = 0.0003 for indicated comparisons). Together, these data demonstrate that DA-mediated induction of *Gadd45b* requires DRD1 signaling through MAPK and CREB signaling pathways.

To determine whether cocaine-related induction of *Gadd45b* in vivo might also occur in a DRD1-specific manner, we next mined a recently published single-nucleus RNA-seq dataset of NAc tissue taken 1 hr after acute cocaine administration [35]. Separate analysis of Drd1-MSNs and Drd2-MSNs identified in this data set revealed cell-selective changes in *Gadd45b* mRNA following cocaine (Fig. S1). Specifically, whereas *Gadd45b* mRNA was increased in Drd1-MSNs following cocaine experience (Mann–Whitney test vs. saline control, *U* = 885173, *p* = 0.0002), it was not significantly induced in Drd2-MSNs (*U* = 467152, *p* = 0.098). These results support the interpretation that DA and cocaine increase *Gadd45b* through DRD1 receptor signaling.

### Knockdown of striatal *Gadd45b* results in dysregulation of transcriptional and DNA methylation dynamics

**Fig. 4:**
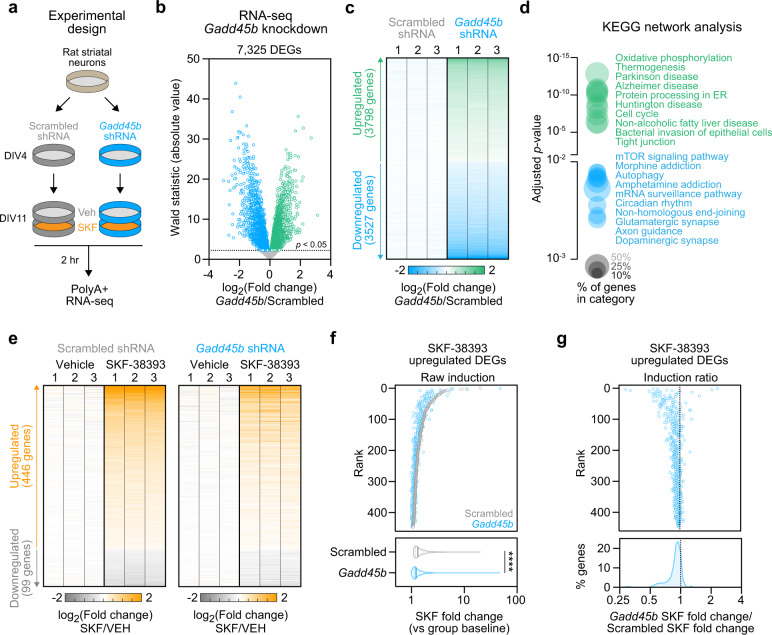
*Gadd45b* knockdown causes widespread transcriptional dysregulation and blocks dopamine-dependent gene programs in striatal neurons. **a** Illustration of experimental design for shRNA-mediated *Gadd45b* knockdown and transcriptional profiling. **b** Volcano plot of 7325 differentially expressed genes (DEGs) in *Gadd45b* shRNA group as compared with scrambled shRNA control group. **c** Heatmap showing replicate values of all DEGs (3798 upregulated, 3527 downregulated) after *Gadd45b* knockdown. **d** KEGG network analysis reveals differential upregulation of genes implicated in oxidative phosphorylation and neurodegenerative disease categories, and downregulation of genes implicated in dopaminergic synapse function and addiction categories. **e** Heatmap of DEGs altered by 2 hr stimulation with the DRD1 receptor agonist SKF-38393 (1 µM), stratified by shRNA treatment group. **f**–**g** Genes upregulated by SKF-38393 stimulation are significantly less induced under conditions of *Gadd45b* knockdown. ****p < 0.0001 for indicated comparisons.

*Gadd45b* is involved in many cellular processes, including those related to learning and memory, neurodevelopment, and cellular stress [28, 29, 44–47]. To further explore the molecular roles of *Gadd45b* in vitro, we designed a custom short-hairpin RNA (shRNA) interference approach to reduce *Gadd45b* mRNA. Lentiviral transduction with *Gadd45b* shRNA produced >90% knockdown of *Gadd45b* mRNA without alterations in mRNA from the control housekeeping gene *Gapdh* (Fig. S2; one-way ANOVA, *F*_(2,29)_ = 66.82, *R*^*2*^ = 0.82, *p* < 0.0001; Tukey’s post hoc tests, *p* < 0.0001 for *Gadd45b* shRNA compared with scrambled shRNA). Bulk RNA-seq of striatal cell cultures identified 7325 DEGs following *Gadd45b* knockdown, consisting of 3527 downregulated genes and 3798 upregulated genes (Fig. 4a–c; Table S2; adjusted *p* < 0.05). KEGG network analysis revealed that genes involved in addiction, as well as DA and glutamatergic synapses, were significantly downregulated following *Gadd45b* knockdown (Fig. 4d, adjusted *p* < 0.05 for all categories shown). In order to characterize which gene programs are active following DRD1 activation, we treated striatal neurons with 1 µM SKF-38393. Compared with scrambled controls, neurons transduced with *Gadd45b* shRNA exhibited a dampened transcriptional response (Fig. 4e; Table S3). Interestingly, many of the genes that are most highly upregulated following DRD1 stimulation in control neurons were blunted in SKF-treated neurons transduced with *Gadd45b* shRNA (Fig. 4e–g; ratio paired *t* test for scrambled vs *Gadd45b* shRNA comparison of SKF-38393 fold change, *t*_(445)_ = 13.57, *p* < 0.0001, *R*^*2*^ = 0.29). Importantly, direct comparison of transcript counts from SKF-38393 upregulated genes demonstrated that *Gadd45b* shRNA did not significantly alter the baseline expression these genes (vs. scrambled control; Wilcoxon matched pairs signed rank test, *p* = 0.2355). This result suggests that the changes produced by *Gadd45b* shRNA were likely the result of a lack of gene induction by SKF-38393, rather than an increase in the baseline expression for these genes. Together, these findings suggest that *Gadd45b* knockdown results in extensive transcriptional dysregulation and prevents the full activation of DA-responsive gene programs in striatal neurons.

Given that *Gadd45b* has previously been implicated in DNA methylation dynamics in the nervous system [22, 23, 48–50], we next explored whether *Gadd45b* knockdown altered DNA methylation in striatal neuron cultures. Using RRBS, we profiled ~1.86 million unique CpG sites following *Gadd45b* shRNA (or scrambled shRNA control) in combination with DRD1 receptor stimulation (vehicle or 1 µM SKF-38393 treatment, *n* = 3/group; Fig. S3a). As expected, DNA methylation patterns exhibited typical bimodal distribution of CpG methylation, with depletion at CpG islands and gene promoters and CpG methylation enrichment within gene bodies (Fig. S3b–e). Surprisingly, *Gadd45b* shRNA did not alter global DNA methylation profiles, as cells with *Gadd45b* knockdown displayed nearly identical DNA methylation levels at regulatory elements, promoters, and gene bodies (Fig. S3c–e). Further, only 129 CpGs (0.006% of CpGs) were differentially methylated (termed dmCpGs) after *Gadd45b* knockdown (Table S4), suggesting largely intact global and site-specific DNA methylation landscapes.

To examine stimulus-induced changes in DNA methylation, we quantified SKF-38393-dependent changes in DNA methylation in the scrambled shRNA control group, and identified 1930 dmCpGs following SKF-38393 treatment as compared with vehicle-treated controls (Fig. S3f; Table S5; *p* < 0.01, >20% change). Notably, SKF-38393-mediated changes in CpG methylation were absent following *Gadd45b* knockdown (Fig. S3f–j), suggesting that *Gadd45b* is necessary for DRD1-dependent changes in DNA methylation. Surprisingly, while *Gadd45b* has been demonstrated to contribute largely to activity-induced DNA hypomethylation, *Gadd45b* shRNA produced robust deficits in both CpG hypermethylation (Wilcoxon matched pairs signed rank test, *W* = 449776, *p* < 0.0001) and hypomethylation (Wilcoxon matched pairs signed rank test, *W* = −481671, *p* < 0.0001) events following SKF-38393 exposure (Fig. S3h–j). Taken together with RNA-seq results, these findings demonstrate that *Gadd45b* is required for key transcriptional and epigenetic alterations downstream of DA receptor activation.

### *Gadd45b* knockdown alters striatal neurophysiology

**Fig. 5:**
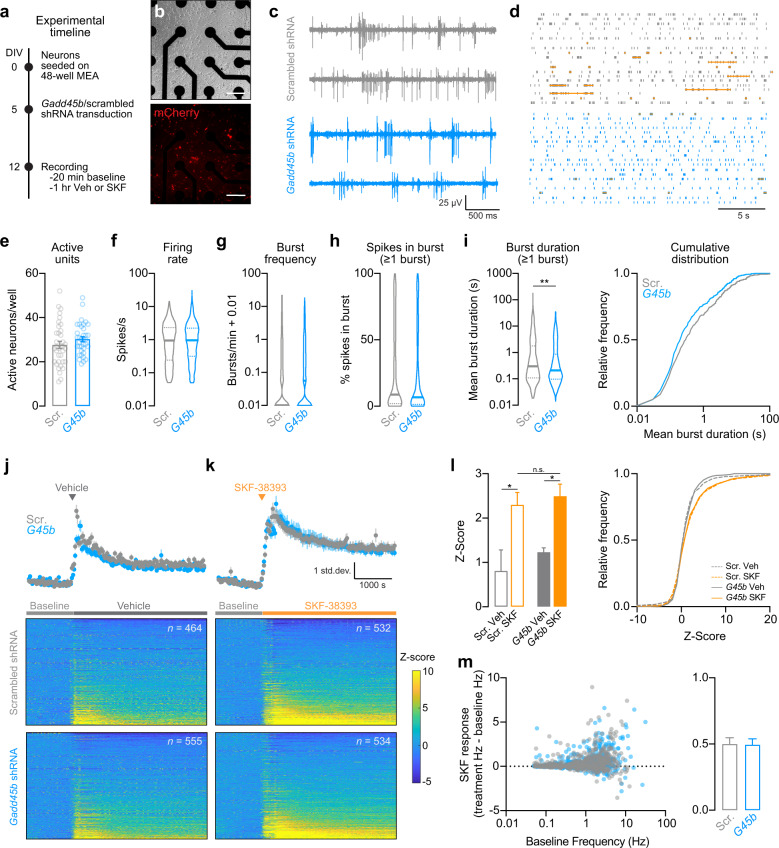
shRNA knockdown of *Gadd45b* in primary striatal neurons alters burst duration, without affecting other electrophysiological characteristics or response to DRD1 stimulation. **a** Experimental timeline for viral transduction and extracellular single-unit recordings. Primary striatal neurons were grown on multielectrode arrays (MEAs) and transduced with lentiviral shRNA constructs on DIV5. **b** Live cell imaging of transduced neurons (expressing mCherry) on DIV13 after collecting electrophysiological measures. Scale bar = 100 μm. **c** Representative traces of four units transduced with either scrambled shRNA or *Gadd45b* shRNA. **d** Representative raster plots (one neuron per row). Orange horizontal lines denote action potential bursts. **e** *Gadd45b* knockdown does not affect the number of spontaneously active units per well. **f**–**g** Action potential frequency and burst frequency are not different between neurons transduced with *Gadd45b* shRNA and scrambled controls. **h** For all neurons with at least one burst, *Gadd45b* knockdown does not alter % of spikes occurring in a burst. **i** shRNA knockdown of *Gadd45b* significantly decreases burst duration compared with scrambled controls. **j**–**k** Mean *Z* score (top) and individual neuron heatmap (bottom) following application of vehicle or 1 µM SKF-38393. **l**, SKF-38393 increases neuronal firing rate as compared with vehicle control. Effects of SKF-38393 did not differ based on *Gadd45b* knockdown status. **m** Left, SKF-38393 response as a function of baseline (pre-SKF-38393) action potential frequency. Right, SKF-38393 response did not differ in *Gadd45b* shRNA treatment group. All bar graphs expressed as mean ± s.e.m. All violin plots expressed as median ± quartiles. *p < 0.05 and **p < 0.01 for indicated comparisons.

Given the large-scale downregulation of genes related to glutamatergic and dopaminergic synaptic function following *Gadd45b* knockdown (Fig. 4d), we next sought to determine whether *Gadd45b* manipulation alters physiological properties of striatal neurons. We transduced striatal cell cultures with shRNA lentiviral constructs prior to electrophysiological characterization using a high-throughput multielectrode array system (Fig. 5a–d). Following transduction on DIV5, we verified expression of shRNA constructs by visualizing mCherry expression (Fig. 5b). On DIV12, we performed a 20 min baseline extracellular electrophysiological recording, then treated cells with either vehicle or SKF-38393 (1 µM) and recorded activity for an additional 1 hr. At baseline, neurons transduced with *Gadd45b* shRNA did not differ from scrambled controls in the number of spontaneously active units (*n* = 996–1089 units across 36 wells/group), mean firing rate, mean action potential burst frequency, or percent of spikes occurring in a burst (Fig. 5e–h). However, we found a significant reduction in burst duration in *Gadd45b* knockdown neurons (*n* = 504 units) as compared with scrambled controls (*n* = 465 units; Fig. 5i, Mann–Whitney *U* test, *U* = 105010, *p* = 0.0052). Application of DRD1 agonist SKF-38393 increased action potential firing rate in both scrambled and *Gadd45b* shRNA neurons, compared with vehicle-treated wells (Fig. 5j–l; one-way Kruskal–Wallace ANOVA, *F* = 12.43, *p* = 0.0061; Dunn’s multiple comparisons test, *p < 0.05 for indicated comparisons). When examining SKF-38393 response as a function of baseline action potential firing rate, we found no difference in SKF-38393 response between the *Gadd45b* knockdown group and scrambled controls, and this lack of effect was maintained across all observed baseline frequencies (Fig. 5m). Thus, *Gadd45b* knockdown significantly reduces burst duration in striatal neurons, without affecting other baseline electrophysiological characteristics or response to DRD1 receptor activation.

## Discussion

Here, we present evidence that *Gadd45b* is an IEG induced in striatal neurons following acute cocaine exposure in vivo and DA treatment in vitro. Furthermore, *Gadd45b* mRNA is upregulated following exposure to cocaine-paired contexts and is necessary for cocaine CPP. In cultured striatal neurons, we demonstrate that *Gadd45b* induction requires DRD1 activation and MAPK signaling cascades. CREB signaling is likely also necessary for *Gadd45b* induction, however CREB inhibition also reduces baseline expression of *Gadd45b*, limiting our interpretation of these findings. Knockdown of *Gadd45b* in striatal neurons blocks the expression of DRD1-responsive genes and decreases the expression of genes implicated in addiction and dopaminergic signaling. In addition, DRD1-mediated bidirectional DNA methylation changes are also inhibited by *Gadd45b* knockdown, irrespective of initial methylation status. Finally, we demonstrate that *Gadd45b* knockdown significantly decreased the duration of action potential bursts in striatal neurons, without altering other electrophysiological characteristics. Interestingly, although *Gadd45b* knockdown blocked DRD1-mediated changes in gene expression and DNA methylation, striatal neurons are able to maintain increased action potential firing following DRD1 activation regardless of *Gadd45b* knockdown status.

### IEG action and transcriptional dynamics of *Gadd45b*

Originally termed *MyD118*, *Gadd45b* was first characterized as a primary response gene important in the myeloid differentiation program [44, 45, 51]. *Gadd45b* is broadly induced by a variety of genotoxic and environmental stimuli, including DNA-alkylating agents [45–47], UV radiation [45–47, 52], anisomycin treatment [45], and exposure to a hyperosmotic environment [45]. In the hippocampus, *Gadd45b* mRNA is increased following contextual fear conditioning [29], electroconvulsive seizure [24, 53], spatial exploration of a novel environment [24], exercise [24], and kainic acid treatment [54–56]. Furthermore, activity-dependent *Gadd45b* induction in hippocampal neurons is dependent on the *N*-methyl-d-aspartate receptor, calcium, and calcium/calmodulin-dependent protein kinase signaling, suggesting that *Gadd45b* is induced in the same manner as classic IEGs, such as *Arc* [24]. In addition, seizure-induced increases in *Gadd45b* are CREB-dependent [54]. *Gadd45b* has also been shown to act as an IEG in other brain regions. For instance, increases in *Gadd45b* mRNA are observed in the NAc after both optical stimulation of VTA DA neuron terminals in the NAc and following direct infusion of brain-derived neurotrophic factor (BDNF) into the NAc [25]. Here, we demonstrate that *Gadd45b* acts as an IEG in the ventral striatum following cocaine administration in vivo (Fig. 1b, f) and DRD1 activation in vitro (Fig. 3c–e), and we show for the first time that striatal induction of *Gadd45b* is dependent on both MAPK and CREB signaling (Fig. 3g–h). Furthermore, we demonstrate that striatal neurons are still capable of physiological responses to DRD1 stimulation following *Gadd45b* knockdown (Fig. 5j–m). This finding suggests that the primary deficit in neurons lacking *Gadd45b* is the failure to translate neuronal activity into a programmed transcriptional and epigenetic response. In addition, the relative lack of change in many neuronal physiological properties following *Gadd45b* manipulation (Fig. 5e–h) indicates that *Gadd45b*-mediated transcriptional reorganization was not associated with overt changes in neuronal phenotype, altered neuronal health, or overall cell death.

### *Gadd45b* in learning and memory processes

Although *Gadd45b* has been previously investigated in the context of learning and memory, its role in these processes remains unclear. Increased *Gadd45b* expression has been observed following the induction of long-term potentiation (LTP), a cellular correlate of learning and memory, alongside other genes with well-established roles in neural plasticity [55, 56]. However, following a near-threshold stimulus, hippocampus slices from *Gadd45b*^*−/−*^ mutant mice exhibit enhanced late-phase LTP, suggesting a plasticity-repressive role of *Gadd45b* [28]. Similarly, studies examining long-term memory in *Gadd45b*^*−/−*^ mutant mice have produced conflicting results. Sultan et al. [28] found that knockout mice displayed enhanced coordination and balance on the accelerating rotarod, increased freezing following hippocampus-dependent contextual fear conditioning, and improved performance during the Morris water maze memory probe, together suggesting that *Gadd45b* may be negatively correlated with motor performance, fear learning, and spatial memory, respectively. In contrast, studies by Leach et al. [29] demonstrated decreased freezing in *Gadd45b*^*−/−*^ mice following contextual fear conditioning, proposing that *Gadd45b* plays an important role in hippocampus-dependent memory processes. In both studies, *Gadd45b* manipulation had no effect on amygdala-dependent cued fear conditioning [28, 29]. Whereas earlier studies involved transgenic knockout mice, the present study expands upon previous findings by implementing non-constitutive, brain region-specific knockdown of *Gadd45b* in the rat striatum using shRNA and CRISPR/Cas9 lentiviral vectors. The data presented here support a pro-memory role of *Gadd45b* in the striatum, with increases in *Gadd45b* mRNA following acute cocaine reward or exposure to a cocaine-paired environment (Fig. 1f). In addition, transgenic *Gadd45b*^*−/−*^ mice and adult rats with NAc-specific CRISPR/Cas9-mediated *Gadd45b* knockdown both exhibit drug-related memory deficits following cocaine-paired place conditioning (Fig. 2). Furthermore, we demonstrate that *Gadd45b* knockdown results in differential upregulation of genes implicated in neurodegenerative diseases, such as Alzheimer’s disease (Fig. 4d).

### Role of *Gadd45b* in DNA methylation dynamics

DNA methylation is a potent epigenetic regulatory modification that is critical for the function and information storage capacity of neuronal systems. In the brain, activity-dependent changes in DNA methylation are central regulators of synaptic plasticity and memory formation and have been implicated in a broad range of neuropsychiatric disease states, including drug addiction. Previous studies have established that *Gadd45b* is required for activity-induced DNA demethylation in the hippocampus, specifically at CpG sites within regulatory regions of *Bdnf* and fibroblast growth factor-1 (*Fgf-1*), genes known to be important for adult neurogenesis in the dentate gyrus [24]. Although no differences were observed in basal methylation levels within these gene regulatory regions in *Gadd45b* knockout mice, activity-induced DNA demethylation at these sites was nearly eliminated. In human post-mortem cortical tissue, decreased GADD45B binding to the *BDNF* promoter was associated with higher levels of 5mC and 5hmC [50]. In the mouse NAc, downregulation of *Gadd45b* alters DNA methylation in a phenotype-, gene-, and locus-specific way, potentially underlying susceptibility or resilience to stress [25]. Despite evidence suggesting that *Gadd45b* is important in activity-dependent DNA demethylation, it is still unclear how the GADD45B protein is involved in this process. GADD45B is an 18-kDa protein that belongs to the ribosomal protein L7Ae/L30e/S12e/Gadd45 superfamily and is known to interact with nuclear hormone receptors and bind nucleic acids [48, 57]. GADD45B contains two LXXLL motifs, domains that are commonly found in other transcriptional coactivators and appear to be required for GADD45B to act as a transcriptional regulator [57].

Several prior reports have revealed locus-specific changes in DNA methylation following cocaine experience [20], as well as a global decrease in NAc methylcytosine following cocaine self-administration and reinstatement [14]. Here, we examined DNA methylation profiles using RRBS following *Gadd45b* knockdown in striatal neurons. We demonstrate that baseline DNA methylation landscapes are preserved following *Gadd45b* knockdown (Fig. S3a–e), but activity-induced changes in CpG methylation following DRD1 activation were absent in neurons lacking *Gadd45b* (Fig. S3f). These results are consistent with prior studies, and suggest that *Gadd45b* is required for Drd1-dependent alterations in DNA methylation. Furthermore, although the above studies have implicated *Gadd45b* in activity-dependent DNA demethylation, we find that *Gadd45b* knockdown blocks both CpG hypermethylation (Fig. S3g, h) and hypomethylation (Fig. S3i, j) following treatment with SKF-38393. Although surprising, it is also important to note several caveats. For example, the RRBS approach used here cannot distinguish DNA methylation and hydroxymethylation. Thus, Drd1-mediated changes in DNA methylation may also reflect changes in hydroxymethylcytosine, which is enriched in the brain and opposes classic effects of methylcytosine [58]. Nevertheless, the observation that *Gadd45b* is required for both increases and decreases in DNA methylation may suggest that this protein functions upstream of signaling mechanisms that direct the genomic precision of DNA methylation changes following neuronal stimulation. Finally, acute cocaine administration in vivo did not induce other genes known to be involved in DNA methylation processes in the NAc after 1 or 24 hr (Fig. 1b). Although our results are largely consistent with the known function of *Gadd45b* in stimulus-regulated DNA methylation changes, future experiments will be required to better characterize the molecular interactions that link *Gadd45b* to this process.

### Influence of *Gadd45b* on striatal signaling and physiology

The NAc is a central integrator of the mesocorticolimbic DA system [59], and many drugs of abuse (and to a lesser extent, natural rewards) increase DA concentrations in this brain region [7, 60]. The NAc is primarily composed of MSNs, which are subclassified into two subpopulations based on the DA receptor expression [61]. DRD1 receptors, expressed on Drd1-MSNs, have a low affinity for DA and are activated by the high concentrations of DA released during bouts of phasic bursting of DA neurons [62, 63]. Pharmacological activation of DRD1 receptors alone is sufficient to produce reward [64], and optogenetic stimulation of Drd1-MSNs promotes positive reinforcement and strengthens associations between cocaine and reward context [65, 66]. Conversely, stimulation of Drd2-MSNs results in aversion and attenuates cocaine reward [65, 66]. Furthermore, in vivo calcium (Ca^2+^) imaging reveals that a single cocaine injection is sufficient to increase Ca^2+^ transient frequency specifically in Drd1-MSNs, while reducing activity in Drd2-MSNs [42]. These physiological responses are also elicited when animals enter a cocaine-associated context, suggesting that these cocaine-associated contextual cues acquire some rewarding properties in themselves, independent of acute cocaine reward [42]. Chemogenetically inhibiting Drd1-MSN activity not only abolishes cocaine-induced increases in Drd1-MSN Ca^2+^ transients, but also blocks the expression of a cocaine place preference [42]. This agrees with recent work from our laboratory demonstrating that acute cocaine experience preferentially activates a subset of Drd1-MSNs in both male and female rats [35]. Here, we demonstrate that *Gadd45b* is upregulated following acute cocaine reward and exposure to a cocaine-paired context (Fig. 1f), and that these increases in *Gadd45b* mRNA are dependent on DRD1 activation and signaling (Fig. 3e–h).

Using high-throughput electrophysiological approaches to record the activity of more than a thousand neurons in vitro, we observed that DRD1 receptor stimulation produced a significant increase in action potential frequency of striatal neurons (Fig. 5j–l). This finding is consistent with recent discoveries that DA produces a rapid but sustained increase in excitability of Drd1-MSNs [67], as well as with prior results demonstrating a key role for DA in synaptic plasticity in this neuronal population [68]. Surprisingly, despite resulting in large-scale changes in gene expression, knockdown of *Gadd45b* did not significantly alter physiological changes in response to the DRD1 agonist SKF-38393. Instead, we observed that *Gadd45b* shRNA produced a relatively specific decrease in the duration of action potential burst events, without altering other electrophysiological parameters such as burst frequency, action potential frequency, or the number of spontaneously active neurons (Fig. 5e–i). Although many factors contribute to action potential burst parameters, it is intriguing to note that RNA-seq analysis following *Gadd45b* knockdown revealed decreases in genes that contribute dopaminergic, glutamatergic, and GABAergic synapse functions. Downregulated genes in these functional categories included many channels or receptors that may alter action potential burst duration, including inwardly rectifying potassium channels (*Kcnj3*, *Kcnj6*, and *Kcnj9*), voltage-gated sodium channels linked to epilepsy (*Scn1a*), ionotropic glutamate receptors (e.g., *Gria3*, *Grik1*, *Grin1*), and GABA receptor subunits (e.g., *Gabbr2*, *Gabra5*, *Gabrb3*, *Gabrg1*, *Gabrg3*).

In vivo, burst firing events in MSNs are regulated by feedforward inhibition from local fast-spiking interneurons, and these events are especially critical for striatum-dependent learning and plasticity [69]. In freely moving rats, burst firing of MSNs in the NAc is temporally associated with important behavioral and reward-related events, including presentation of reward-paired cues [70, 71], delivery/consumption of natural and drug rewards [71–73], and operant responses for rewarding stimuli [74–76]. Importantly, these phasic increases in neuronal activity encode key aspects of reward value and cost [77–79] and are thought to contribute to the key role of the NAc in associative learning and addiction [80, 81]. Consistent with this role, we demonstrate that complete knockout or NAc-specific knockdown of *Gadd45b* also results in impaired cocaine place learning. Future studies will be required to identify how *Gadd45b* contributes to specific learning-related activity of NAc neurons in the context of drug reinforcement.

## Conclusions and future directions

In the current study, we describe a role for striatal *Gadd45b* in DA-dependent transcriptional regulation and cocaine reward, suggesting that *Gadd45b* is involved in the development and maintenance of addiction. These findings build upon previous work establishing a role for *Gadd45b* in neurodevelopmental disorders and neuropsychiatric disease states, including major psychosis [50], depression [25], and autism spectrum disorder [82]. Together, these results highlight that *Gadd45b* is dysregulated in the pathophysiology of other mental health conditions. Although the underlying mechanisms of its involvement may differ by cell type and brain region, a thorough understanding of *Gadd45b*-dependent regulation of transcriptional and epigenetic dynamics will be an important next step for revealing potential therapeutic avenues targeting this pathway.

## Funding and disclosure

This work was supported by NIH grants DP1-DA039650, R00-DA034681, and R01-MH114990 (JJD), F32-DA041778 (FAS), and T32-GM008361, and T32-GM008111 (MEZ). LI is supported by the Civitan International Research Center at UAB. Additional assistance to JJD was provided by the UAB Pittman Scholars Program.

## Supplementary information

Supplemental Materials

Figure S1

Figure S2

Figure S3

Table S1

Table S2

Table S3

Table S4

Table S5
