# Supplementary material for: Regulation of dopamine-dependent transcription and cocaine action by Gadd45b
Source: Neuropsychopharmacology. 2020 Sep 14;46(4):709–20. doi: 10.1038/s41386-020-00828-z (PMC8027017; doi:10.1038/s41386-020-00828-z)
Supplement: Supplementary file 1 — Supplemental Materials [file 41386_2020_828_MOESM1_ESM.docx]

**Supplemental Materials and Methods**

**Regulation of dopamine-dependent transcription and cocaine action by *Gadd45b***

Morgan E. Zipperly^1^, Faraz A. Sultan^1^, Guan-En Graham^1^, Andrew C. Brane^1^, Natalie A. Simpkins^1^, Nancy V.N. Carullo^1^, Lara Ianov^2^, and Jeremy J. Day^1,2^*

**Supplemental Figures**

**Figure S1.** Cocaine-induced changes in *Gadd45b* mRNA are specific to Drd1-MSNs.

**Figure S2.** Efficient gene knockdown using RNA interference in primary striatal neuron cultures.

**Figure S3.** *Gadd45b* knockdown prevents dopamine-driven DNA methylation changes.

**Supplemental Tables**

**Table S1.** Sequences of PCR primers, CRISPR sgRNAs, and shRNA targets.

**Table S2.** Differentially expressed genes following *Gadd45b* shRNA.

**Table S3.** Differentially expressed genes following SKF-38393 (1 µM).

**Table S4.** Differentially methylated CpGs following *Gadd45b* shRNA.

**Table S5.** Differentially methylated CpGs following SKF-38393 (1 µM).

**Supplemental Methods**

***Animals.*** All experiments were performed in accordance with the University of Alabama at Birmingham Institutional Animal Care and Use Committee. Sprague-Dawley timed pregnant dams and adult male rats (90-120 days old) were purchased from Charles River Laboratories (Wilmington, MA, USA). Dams were individually housed until embryonic day 18 (E18) for cell culture harvest. Male adult rats were co-housed in pairs in plastic filtered cages with nesting enrichment in an AAALAC-approved animal care facility maintained between 23-24°C on a 12 hr light/dark cycle with ad libitum food (Lab Diet Irradiated rat chow) and water. Bedding and enrichment were changed weekly by animal resources program staff. Animals were randomly assigned to experimental groups. *Gadd45b* knockout mice for the cocaine conditioned place preference experiment were bred at the University of Alabama at Birmingham on a B6:129VJ background, as described previously [1,2]. Male wild-type and mutant offspring from heterozygotes were used for behavioral testing. Mice aged 2-6 months were individually housed at least 3 days prior to the start of behavioral testing. Heterozygotes were bred with C57BL/6 wild-types for at least six generations to generate back-crossed mice. All animals were handled for 3-5 days prior to behavioral testing.

***Drugs.*** Cocaine hydrochloride (C5776, Sigma-Aldrich, St. Louis, MO, USA) was dissolved in sterile 0.9% sodium chloride and injected intraperitoneally (i.p.) at a dose of 10 mg/kg or 20 mg/kg for cocaine locomotor sensitization and conditioned place preference testing. Cocaine solution was made fresh immediately before behavioral testing and was protected from light.

For *in vitro* experiments, drugs were diluted in Neurobasal medium (Invitrogen, Waltham, MA, USA) in a sterile hood immediately prior to treating cultured neurons. Dopamine hydrochloride (1 µM; H8502, Sigma-Aldrich) was dissolved in Neurobasal medium. R(+)-SCH-23390 hydrochloride (D054, Sigma-Aldrich) and R(+)-SKF-38393 hydrochloride (S101, Sigma-Aldrich) were dissolved in sterile Milli-Q water and diluted to 1 µM in Neurobasal medium. (-)-Quinpirole hydrochloride (Q102, Sigma-Aldrich) was dissolved in sterile Milli-Q water and diluted to 1 µM in Neurobasal medium. Forskolin, 7-Deacetyl-7-[O-(N-methylpiperazino)-gamma-butyryl]-dihydrochloride (20 µM; 344273, EMD Millipore, Billerica, MA, USA) was dissolved in sterile Milli-Q water, then diluted in Neurobasal medium. For MEK inhibitor experiments, U0124 (Millipore, 662006) and U0126 (662005, Millipore) were dissolved in DMSO (D12345, Invitrogen) and diluted with Neurobasal medium to 1 µM. The CREB inhibitor 666-15 (5661, Tocris, Minneapolis, MN, USA), also called 3-(3-Aminopropoxy)-N-[2-[[3-[[(4-chloro-2-hydroxyphenyl)amino]carbonyl]-2-naphthalenyl]oxy]ethyl]-2-naphthalenecarboxamine hydrochloride, was dissolved in DMSO, and cells were treated at a dose of 1 µM.

***Neuronal cell cultures.*** Primary rat striatal cell cultures were generated from E18 striatal tissue as described previously [3–5]. Cell culture plates (Denville Scientific, Inc., Plainfield, NJ, USA) were coated overnight with poly-L-lysine (50 µg/mL; Sigma-Aldrich), supplemented with 7.5 µg/mL laminin (Sigma-Aldrich), and rinsed with diH_2_O. Multielectrode arrays (MEAs; Axion Biosystems, Atlanta, GA, USA) were coated with polyethyleneimine (Sigma-Aldrich). Dissected striatal tissue was incubated with papain (LK003178, Worthington Biochemical Corporation, Lakewood, NJ, USA for 25 min at 37°C. After rinsing in complete Neurobasal media (supplemented with B27 and L-glutamine, Invitrogen), a single-cell suspension was prepared by sequential trituration through large to small fire-polished Pasteur pipettes and filtered through a 100 µm cell strainer (Fisher Scientific, Waltham, MA, USA). Cells were pelleted, re-suspended in fresh media, counted, and seeded to a density of 125 000 cells per well on 24-well culture plates (65 000 cells/cm^2^) or 30 000 cells per well on 48-well MEA plates. Cells were grown in complete Neurobasal media for 12 days in vitro (DIV12) in a humidified CO_2_ (5%) incubator at 37°C with half-media changes at DIV1, 4-5, and 8-9. MEAs received a one-half media change to BrainPhys (Stemcell Technologies Inc., Vancouver, BC, Canada) with SM1, L-glutamine supplements, and penicillin-streptomycin starting on DIV4-5 and continued every 3-4 days.

***RNA extraction and RT-qPCR.*** Total RNA was extracted (RNAeasy kit, Qiagen, Hilden, Germany) and reverse-transcribed (iScript cDNA Synthesis Kit, Bio-Rad, Hercules, CA, USA). cDNA was subject to RT-qPCR for genes of interest, as described previously [4,5]. A list of PCR primer sequences is provided in **Supplemental Table S1**.

***Tissue collection from adult NAc.*** For RNA sequencing and RT-qPCR experiments, animals were rapidly decapitated either 1 hr or 24 hr after i.p. injection of either 10 mg/kg cocaine or saline on the final day of testing. Brain tissue was extracted bilaterally and immediately frozen on dry ice. Tissue was stored at -80°C until the day of sequencing or RNA isolation and subsequent RT-qPCR. For CPP experiments, rats were deeply anesthetized with 4-5% isoflurane, then transcardially perfused with formalin (1:10 dilution in PBS, Fisher). Brains were removed and post-fixed for 24 hr in formalin at 4°C, protected from light. Fixed tissue was then rinsed and stored in 1xPBS at 4°C until sliced at 50 µm using a vibratome. Slices were mounted on glass microscope slides with Prolong Gold anti-fade medium (Invitrogen) containing 4,6-diamidino-2-phenylindole (DAPI) stain as a marker for cell nuclei and coverslipped. Bilateral viral expression and NAc placement were verified manually using a Nikon TiS inverted fluorescent microscope and/or a Zeiss Airyscan LSM800 confocal microscope (for representative image in **Figure 2**).

***CRISPR/Cas9 and RNAi construct design***. CRISPR and shRNA constructs for editing or knockdown of *Gadd45b* were delivered using second-generation lentiviral expression vectors. Cas9 and CRISPR sgRNAs were expressed using a modified version of the lentivirus compatible expression vector lentiCRISPR v2 [6], which was a gift from Feng Zhang (Addgene plasmid #52961; http://n2t.net/addgene:52961; RRID: Addgene_52961). The puromycin resistance cassette in the lentiCRISPR v2 backbone was replaced with a mammalian codon-optimized EGFP tag using MluI and BamHI restriction digest. *Gadd45b*-specific sgRNA targets were designed using online tools provided by the German Cancer Research Center (http://www.e-crisp.org/E-CRISP/). To ensure specificity, CRISPR RNA (crRNA) sequences were analyzed with Cas-OFFinder [7]. Critically, the *Gadd45b* crRNA sequence did not have any identical matches elsewhere in the rat genome, had no single base mismatches, and had only one two-nucleotide mismatch sequence, which was not located in a gene (**Supplemental Table S1**).

Short hairpin RNAs were designed using the Broad Institute Genetic Perturbation Platform web portal. A pLKO.1-TRC vector (a gift from David Root; Addgene plasmid #10879; http://n2t.net/addgene:10879; RRID:Addgene_10879) [8] was cloned into an expression vector with an mCherry reporter (Addgene plasmid #114199) [3] to generate distinct U6-shRNA and EF1α-mCherry expression cassettes. Oligonucleotides containing the shRNA sequence were cloned into this backbone using AgeI and EcoRI restriction digest. A list of the sgRNA and shRNA target sequences is provided in **Supplemental Table S1**.

***Lentivirus production.*** Viruses were made as described previously [3,5]. All viruses were produced under sterile BSL-2 conditions by transfecting HEK-293T cells (ATCC CRL-3216) with the specified CRISPR or RNAi plasmid, the psPAX2 packaging plasmid, and the pCMV-VSV-G envelope plasmid (Addgene 12260 and 8454) with Fugene HD (Promega, Madison, WI, USA) for 40-48 hr in supplemented Ultraculture media (L-glutamine, sodium pyruvate, and sodium bicarbonate) in a T225 culture flask. Supernatant was passed through a 0.45 µm filter and centrifuged at 106 883 rcf for 1 hr 45 min at 4°C. The viral pellet was resuspended in 1/100^th^ (*in vitro*) or 1/1000^th^ (*in vivo*) supernatant volume of sterile PBS and stored at -80°C. Physical viral titer was determined using either Lenti-X qRT-PCR Titration Kit (Takara, Mountain View, CA, USA) or qPCR Lentivirus Titration Kit (Applied Biological Materials Inc., Richmond, BC, Canada). Viral titers were 1.425 x 10^9^ GC/mL for *lacZ* sgRNA control, 2.663 x 10^9^ GC/mL for *Gadd45b* sgRNA, 2.58 x 10^12^ GC/mL for the *Gadd45b* shRNA, and 7.54 x 10^11^ GC/mL for the scrambled shRNA control. Viruses were stored in sterile PBS at -80°C in single-use aliquots.

***Multi-electrode array recordings.*** Single-unit electrophysiological activity was recorded using an Axion Maestro Pro recording system (Axion Biosystems). E18 rat primary striatal neurons were seeded in 48-well MEAs at 30 000 cells/well, as described above. Each MEA well within the 48-well plate contains 16 extracellular recording electrodes and a ground electrode. Neurons were transduced with RNAi constructs on DIV5 and MEA recordings were performed on DIV12 while connected to a temperature- and CO_2_-controlled system (maintained at 37°C and 5% CO_2_). Cell health and viral mCherry expression were optically verified using a Nikon TiS inverted fluorescent microscope. Electrical activity was measured by an interface board at 12.5 kHz, digitized, and transmitted to an external computer for data acquisition and analysis in Axion Navigator software (v.1.5.1, Axion Biosystems). All data were filtered using dual 0.01 Hz (high pass) and 5 000 Hz (low pass) Butterworth filters. Action potential thresholds were set automatically using an adaptive threshold for each electrode (> 6 standard deviations from the electrode’s mean signal). Neuronal waveforms collected in Axion Navigator software were exported to Offline Sorter (v.4.0, Plexon, Dallas, TX, USA) for sorting of distinct waveforms corresponding to units on individual electrode channels. Waveforms were sorted by the authors and waveform isolation was confirmed using principal component analysis, inter-spike intervals, and auto- or cross-correlograms. Further analysis of burst activity and firing rate was performed in NeuroExplorer software (v.5.0). Burst activity was analyzed using a Poisson burst surprise = 5.

***RNA-sequencing.*** Bulk RNA-sequencing (RNA-seq) was carried out at the UAB Genomics Core Laboratories at the University of Alabama at Birmingham. RNA was extracted, purified (RNeasy, Qiagen), and DNase-treated for three biological replicates per experimental condition. RNA quality was determined on the BioAnalyzer 2100 (Agilent Technologies, Wilmington, DE, USA). RNA sequencing libraries were created using the NEBNext Ultra II Directional RNA-Seq library kit (NEB, Ipswich, MA, USA) according to manufacturer’s recommendations. The resulting libraries underwent sequencing (75 bp paired-end directional reads; 28.9 - 48.7 million reads*/*sample) on an Illumina NextSeq 500 sequencing platform using standard techniques.

***RNA-seq data analysis.*** Paired-end FASTQ files were uploaded to the High Performance Computing cluster at the University of Alabama at Birmingham for custom bioinformatics analysis using a pipeline built with snakemake [9] (v5.1.4). Read quality, length, and composition were assessed using FastQC prior to trimming low quality bases (Phred < 20) and Illumina adapters (Trim_Galore! v04.5). Splice-aware alignment to the Rn6 Ensembl genome assembly (v90) was performed with STAR [10] (v2.6.0c). An average of 89.01% of reads were uniquely mapped. Binary alignment map (BAM) files were merged and indexed with Samtools (v1.6). Gene-level counts were generated using the featureCounts [11] function in the Rsubread package (v1.26.1) in R (v3.4.1), with custom options (isGTFAnnotationFile = TRUE, useMetaFeatures = TRUE, isPairedEnd = TRUE, requireBothEndsMapped = TRUE, strandSpecific = 2, and autosort = TRUE). DESeq2 v 1.16.1 (*72*) in R was used to perform count normalization and differential gene expression analysis with the application of Benjamini-Hochberg false discovery rate (FDR) for adjusted p-values. Differentially expressed genes (DEGs) were designated at adjusted *p* < 0.05 and basemean > 50.

Gene enrichment analysis was performed using the Kyoto Encyclopedia of Genes and Genomes (KEGG) network database (version KEGG_09.04.2019) in the ClueGO v2.3.4 application in Cytoscape, using the protein-coding rat genome as a reference set. Enrichment analysis applied Benjamini-Hochberg correction for multiple comparisons and required a minimum of 3 genes per enriched KEGG term category. Significantly enriched categories were designated using a right-sided hypergeometric test, with adjusted *p* < 0.05.

***Reduced representation bisulfite sequencing (RRBS).*** RRBS was carried out at the Heflin Center for Genomic Science Genomics Core Laboratories at the University of Alabama at Birmingham. Primary striatal cultures were transduced with scrambled or *Gadd45b* shRNA-expressing lentiviruses at DIV4, and stimulated with 1 µM SKF-38393 (or vehicle) for 2 hr at DIV11. Genomic DNA from ~250,000 neurons per sample (*n* = 3 samples per group) was extracted and purified (DNeasy Blood and Tissue DNA extraction kit, Qiagen) prior to RRBS (Ovation RRBS Methyl-Seq System, NuGen, #0353), used according to manufacturer’s instructions. Bisulfite-converted DNA libraries underwent sequencing (75 bp single-end reads; ∼28.7-35.4 million reads/sample) on an Illumina sequencing platform (NextSeq 500).

***RRBS data analysis.*** Single-end FASTQ files were uploaded to the High Performance Computing cluster at the University of Alabama at Birmingham for custom bioinformatics analysis using a pipeline built with snakemake [9] (v5.2.2). Read quality, length, and composition were assessed using FastQC prior to trimming low quality bases and kit-specific diversity trimming (Trim_Galore! v04.5). Alignment to the UCSC Rn6 genome assembly was performed with Bismark v0.19.0 (using Bowtie2 v2.3.4.1 and Samtools v1.6). An average of 70.98% of reads were uniquely mapped, and Bismark coverage files generated for CpG methylation were further analyzed in Seqmonk v1.45.4, with unique samples grouped into replicate sets for analysis. DNA methylation status at individual CpGs was quantitated as forward (methylated) reads/total reads at 1 855 525 million CpG sites having > 120 reads for the entire dataset. Differentially methylated CpGs (dmCpGs) across replicate sets were detected using EdgeR [12] (v3.1), setting statistical significance cutoffs at *p* < 0.01 and methylation change > ± 20%.

***Stereotaxic surgery.*** Naïve adult male Sprague-Dawley rats (Charles River) were anesthetized with 4-5% isoflurane and placed in a stereotaxic apparatus (Kopf instruments, Tujunga, CA, USA). Rats were maintained at a surgical plane of anesthesia with 2-2.5% isoflurane; rats’ respiratory rates were monitored throughout surgery and kept between 35-55 rpm. Surgical coordinates were determined using Paxinos and Watson [13] as a guide to target the NAc core. Under aseptic conditions, guide holes were drilled at AP +1.6 mm, ML ± 1.4 mm, and the infusion needle was lowered to DV -7.0 (all coordinates with respect to bregma). All infusions were made using a gastight 30-gauge stainless steel injection needle and 10 µL syringe (Hamilton Company, Reno, NV, USA). Lentivirus constructs were microinfused at a rate of 0.25 µL/min using a syringe pump (Harvard Apparatus, Holliston, MA, USA), totaling 2 µL per hemisphere. Following each infusion, needles remained in place for 10 min to allow for diffusion of virus. After retracting the infusion needles, guide holes were filled with sterile bone wax and the surgical incision was closed with nylon sutures. Rats received buprenorphine (0.03 mg/kg) and carprofen (5 mg/kg) for pain management and inflammation, as well as topical bacitracin (500 units) to prevent infection at the incision site.

***Acute cocaine locomotor testing and cocaine sensitization***. For open-field locomotor testing, naïve male rats (*n* = 12 per group) were given i.p. injections of saline on days 1 and 2 immediately before 30 min of locomotor testing. On days 3 and 10, half of the rats were given an i.p. injection of 10 mg/kg cocaine, and the other half received i.p. injections of saline. Locomotor activity was monitored in a 43 x 43 cm plexiglass locomotor activity chamber (Med Associates, Inc., St. Albans, VT, USA) with opaque white wall covering and an open top. Each chamber included a 48-channel X-Y infrared array (Med Associates, Inc.) that was used to measure distance traveled in conjunction with Activity Monitor software (Med Associates, Inc.). Test chambers were cleaned with 0.0156% chlorhexidine and 70% ethanol at the beginning and end of each testing day, and cleaned with 70% ethanol in between trials. Animals were transported to the behavioral testing core 30 min prior to testing on a covered cart. Behavioral test sessions were conducted during the light cycle, and the overhead lights and white noise generator remained on in the testing room. The same male experimenter conducted all locomotor testing and was present in the room during testing.

***Conditioned place preference testing.*** Conditioned place preference (CPP) testing was completed in a three-chamber apparatus with guillotine doors (Med Associates, Inc.). The two chambers used for conditioning measured approximately 27 x 21 x 22 cm, one with opaque black walls and stainless steel bar flooring, and the other with opaque white walls and metal wire grid flooring. The conditioning chambers were separated from one another by guillotine-style doors and a central grey compartment with solid flooring, measuring 12 x 21 x 22 cm. All three chambers had clear perforated acrylic lids with house lights. Each apparatus included a 16-channel infrared controller (Med Associates, Inc.) to track rodent position, in conjunction with Med PC software (v4.1.49; Med Associates, Inc.).

For CPP testing in transgenic *Gadd45b* knockout mice, naïve male mice (*n* = 7-9 per group) were placed in the central compartment on the first day of testing (i.e. pre-test) and were permitted to explore all 3 chambers of the CPP apparatus during a 20 min session. On days 2 and 4, mice were given an i.p. injection of saline immediately prior to being placed in the initially preferred chamber for the 20 min conditioning session. On days 3 and 5, mice were given an i.p. injection of 10 mg/kg cocaine before being placed in the initially non-preferred chamber for the 20 min conditioning session. On day 6 (i.e. post-test), mice were again placed in the central compartment and allowed to freely move between all three chambers. Time spent in each chamber during the post-test was calculated and compared to the pre-test times. Overhead lights were off, and CPP house lights were set to an intensity of 10. Cedar and pine beddings were used in the white and black chambers, respectively, to help mice further differentiate between the environments.

CPP testing in adult male rats (*n* = 7-8 per group) began 2 weeks following viral infusion surgeries. Days 1-6 of testing were identical to CPP testing in transgenic mice. However, this schedule was repeated in rats on days 7-10, increasing the cocaine dose to 20 mg/kg for cocaine conditioning on days 8 and 10. On day 11, rats underwent a second post-test to measure cocaine place preference. Overhead lights were left on, and CPP house lights were set to an intensity of 7-8. Testing and conditioning sessions lasted 30 min.

***Statistical analysis.*** Sample sizes were calculated using a freely available calculator [14]. Transcriptional differences from RT-qPCR experiments were compared with an unpaired *t*-test with Welch’s correction, one-way ANOVA with Tukey’s *post hoc* tests, or two-way ANOVA with Sidak’s *post hoc* tests, where appropriate. MEA data were compared with Mann-Whitney *U*-tests. Raw distance travelled data from days 3 and 10 of locomotor testing were compared within groups using paired *t*-tests. Locomotor sensitization data for days 3 and 10 were normalized within each group to mean locomotion of baseline days 1 and 2. Normalized locomotor sensitization data were compared using a two-way ANOVA with Tukey’s *post hoc* tests where appropriate. CPP data were compared with a two-way ANOVA with Bonferroni’s multiple comparisons test. Statistical significance was designated at α = 0.05 for all analyses. Statistical and graphical analyses were performed with Prism software (GraphPad, La Jolla, CA). Statistical assumptions (e.g. normality and homogeneity for parametric tests) were formally tested and examined via boxplots.

***Data availability.*** Sequencing data that support the findings of this study will be deposited at Gene Expression Omnibus. All relevant data that support the findings of this study are available by request from the corresponding author (J.J.D.).

**References**

1. Gupta M, Gupta SK, Balliet AG, Hollander MC, Fornace AJ, Hoffman B, et al. Hematopoietic cells from Gadd45a- and Gadd45b-deficient mice are sensitized to genotoxic-stress-induced apoptosis. Oncogene. 2005;24:7170–7179.

2. Sultan FA, Wang J, Tront J, Liebermann DA, Sweatt JD. Genetic Deletion of gadd45b, a Regulator of Active DNA Demethylation, Enhances Long-Term Memory and Synaptic Plasticity. J Neurosci. 2012;32:17059–17066.

3. Savell KE, Bach SV, Zipperly ME, Revanna JS, Goska NA, Tuscher JJ, et al. A neuron-optimized CRISPR/dCas9 activation system for robust and specific gene regulation. Eneuro. 2019;6:ENEURO.0495-18.2019.

4. Savell KE, Gallus NVN, Simon RC, Brown JA, Revanna JS, Osborn MK, et al. Extra-coding RNAs regulate neuronal DNA methylation dynamics. Nat Commun. 2016;7:12091.

5. Savell KE, Tuscher JJ, Zipperly ME, Duke CG, Phillips RA, Bauman AJ, et al. A dopamine-induced gene expression signature regulates neuronal function and cocaine response. Sci Adv. 2020;6:eaba4221.

6. Sanjana NE, Shalem O, Zhang F. Improved vectors and genome-wide libraries for CRISPR screening. Nat Methods. 2014;11:783–784.

7. Bae S, Park J, Kim J-S. Cas-OFFinder: a fast and versatile algorithm that searches for potential off-target sites of Cas9 RNA-guided endonucleases. Bioinformatics. 2014;30:1473–1475.

8. Moffat J, Grueneberg DA, Yang X, Kim SY, Kloepfer AM, Hinkle G, et al. A Lentiviral RNAi Library for Human and Mouse Genes Applied to an Arrayed Viral High-Content Screen. Cell. 2006;124:1283–1298.

9. Köster J, Rahmann S. Snakemake—a scalable bioinformatics workflow engine. Bioinformatics. 2012;28:2520–2522.

10. Dobin A, Davis CA, Schlesinger F, Drenkow J, Zaleski C, Jha S, et al. STAR: ultrafast universal RNA-seq aligner. Bioinformatics. 2013;29:15–21.

11. Liao Y, Smyth GK, Shi W. featureCounts: an efficient general purpose program for assigning sequence reads to genomic features. Bioinformatics. 2014;30:923–930.

12. Robinson MD, McCarthy DJ, Smyth GK. edgeR: a Bioconductor package for differential expression analysis of digital gene expression data. Bioinformatics. 2010;26:139–140.

13. Paxinos G, Watson C. The rat brain in stereotaxic coordinates. 2009.

14. Lenth RV. Java Applets for Power and Sample Size. 2006.
