# Supplementary material for: Regulation of dopamine-dependent transcription and cocaine action by Gadd45b
Source: Neuropsychopharmacology. 2020 Sep 14;46(4):709–20. doi: 10.1038/s41386-020-00828-z (PMC8027017; doi:10.1038/s41386-020-00828-z)
Supplement: Supplementary file 2 — Figure S1 [file 41386_2020_828_MOESM2_ESM.pdf]

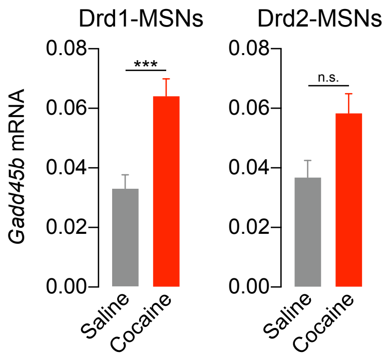

Figure S1. Cocaine-induced changes in *Gadd45b* mRNA are specific to Drd1-MSNs. snRNA-seq dataset from NAc tissue harvested 1 hr following acute cocaine (20 mg/kg; from Savell, Tuscher, Zipperly, Duke, Phillips et al., *Science Advances* 2020). In Drd1-MSNs (saline, n=1151; cocaine, n=1597), *Gadd45b* mRNA is increased by cocaine. In Drd2-MSNs (saline, n=815; cocaine, n=1168), *Gadd45b* does not differ in saline and cocaine treated rats. ***p < 0.001 for indicated comparison.
