# Supplementary material for: Regulation of dopamine-dependent transcription and cocaine action by Gadd45b
Source: Neuropsychopharmacology. 2020 Sep 14;46(4):709–20. doi: 10.1038/s41386-020-00828-z (PMC8027017; doi:10.1038/s41386-020-00828-z)
Supplement: Supplementary file 3 — Figure S2 [file 41386_2020_828_MOESM3_ESM.pdf]

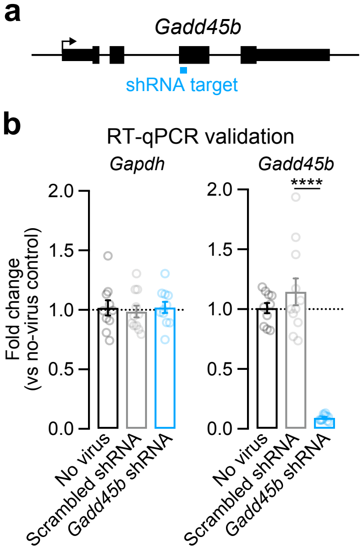

Figure S2. Efficient gene knockdown using RNA interference in primary striatal neuron cultures. **a,** A short hairpin RNA (shRNA) was engineered to target exon 3 of *Gadd45b* and packaged in a lentivirus backbone to drive gene knockdown. **b,** RT-qPCR validation revealed no effect on the housekeeping gene *Gapdh,* but a significant knockdown in *Gadd45b* mRNA compared to no-virus and scrambled shRNA controls. ****p < 0.0001 for indicated comparisons.
