# Supplementary material for: Regulation of dopamine-dependent transcription and cocaine action by Gadd45b
Source: Neuropsychopharmacology. 2020 Sep 14;46(4):709–20. doi: 10.1038/s41386-020-00828-z (PMC8027017; doi:10.1038/s41386-020-00828-z)
Supplement: Supplementary file 4 — Figure S3 [file 41386_2020_828_MOESM4_ESM.pdf]

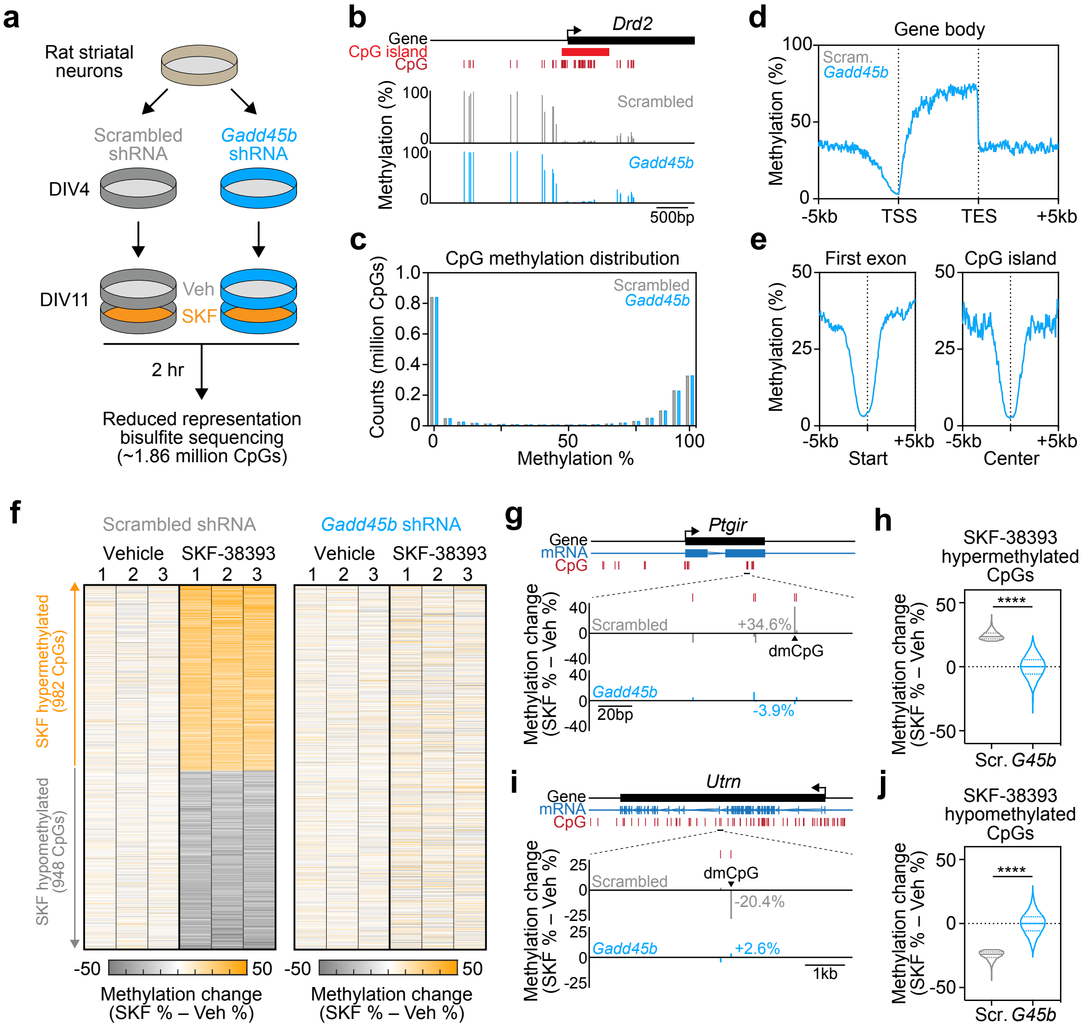

Figure S3. *Gadd45b* knockdown prevents dopamine-driven DNA methylation changes. **a,** Illustration of experimental design. Rat striatal cultures were transduced with *Gadd45b* shRNA (or scrambled control) at DIV4. Cultured neurons were treated with the DRD1 receptor agonist SKF-38393 (1 μM) for 2 hr at DIV11 prior to DNA extraction and genome-wide DNA methylation profiling with reduced representation bisulfite sequencing (RRBS). **b**, RRBS tracks from representative gene locus (*Drd2*) and promoter-spanning CpG island. Only CpGs with > 120 reads are shown. **c**, Genome-wide distribution of CpG methylation values from ~1.86 million CpGs reveals bimodal distribution of CpG methylation in both scrambled and *Gadd45b* shRNA groups. **d**-**e**, DNA methylation profiles across gene bodies, first exon, and CpG islands reveals similar CpG methylation landscapes in scrambled and *Gadd45b* shRNA groups. **f**, Heatmaps showing CpG methylation change (SKF-38393 % - Veh %) for all 1930 CpGs modulated by SKF-38393 in the scrambled shRNA condition (termed differentially methylated CpGs (dmCpGs; defined as $p < 0.01$, > 20% change)). **g**, Representative SKF-38393 hypermethylated dmCpG at the *Ptgir* gene locus. **h**, *Gadd45b* shRNA prevents SKF-38393-induced increases in CpG methylation. **i**, Representative SKF-38393 hypomethylated dmCpG at the *Utrn* gene locus. **j**, *Gadd45b* shRNA prevents decreases in CpG methylation following DRD1 agonist treatment. ****$p < 0.0001$ for indicated comparisons.
